# *Ignatzschineria larvae* bacteriemia in an intensive care unit patient: a case report and a review of the literature

**DOI:** 10.1016/j.idcr.2026.e02625

**Published:** 2026-05-23

**Authors:** Angèle Roudeau, Paul Badoual, Djamal Haouchine, Nicolas Argy, Émilie Rondinaud, Julien Le Marec

**Affiliations:** a Department of Bacteriology, Bichat-Claude Bernard hospital, Assistance Publique – Hôpitaux de Paris, Paris, France; b Intensive Care Unit, Bichat-Claude Bernard hospital, Assistance Publique – Hôpitaux de Paris, Paris, France; c Department of Parasitology, Bichat-Claude Bernard hospital, Assistance Publique – Hôpitaux de Paris, Paris, France; d UMR MERIT IRD 216, Inserm U1344, Faculté de pharmacie de Paris, Université Paris Cité, Paris, France; e UMR 1137, INSERM, IAME, Université Paris Cité and Université Sorbonne Paris Nord, Paris, France

**Keywords:** *Ignatzschineria*, Myiasis, WGS, antibacterial drug resistance, intensive care unit

## Abstract

**Background:**

*Ignatzschineria larvae* is a Gram-negative bacterium found in the microbiota of several fly species but rarely involved in human pathology.

**Case presentation:**

We report the case of a patient admitted to the intensive care unit for sepsis with a bloodstream infection due to *Ignatzschineria larvae* and methicillin-susceptible *Staphylococcus aureus* secondary to a scalp wound infestation by *Calliphora vomitaria*. Morphological examination allowed identification of the larva, bacterial culture and molecular biology techniques identified the bacteria and its antibiotic susceptibility and resistance. An unusual resistance profile, including fluoroquinolones, fosfomycin and cyclines, was observed. NGS identified the underlying genetic determinant. The patient was successfully treated with cefotaxime and daptomycin then trimethoprim-sulfamethoxazole and oxacillin.

**Discussion:**

Only eight *Ignatzschineria larvae* infections in humans had been previously reported, always from a skin barrier alteration in patients with poor social conditions and often associated with chronic alcohol consumption. Although most strains described in the literature are susceptible to beta-lactam antibiotics, our clinical case provides additional documentation necessary for optimizing the management of this type of infection.

## Introduction

*Ignatzschineria larvae* is a Gram-negative bacterium found in the microbiota of several fly species responsible for cutaneous myiasis. Its involvement in human pathology has now been documented with bacteremia caused by *I. larvae* described in patients with wounds infested with larvae, but these cases remain rare (fewer than ten cases in the literature). In this context, we report the case of *I. larvae* bacteremia in a patient admitted to an intensive care unit (ICU), with a phenotypic and a genomic approach for bacteria identification and antibiotic resistance exploration and with an entomological description for myiasis.

## Case presentation

A 55-year-old homeless patient with a history of chronic alcohol intoxication was found disoriented in the street and brought to the emergency department.

The patient’s vital signs were as following: heart rate 119 bpm, blood pressure 92/49 mmHg, respiratory rate 16/min, temperature 37,3°C and Glasgow Coma Scale 7, and he presented mottling of the lower legs. Clinical examination revealed innumerable maggots emerging from a large scalp wound and both auditory canals. Blood tests showed severe metabolic acidosis (pH 7.09, lactate 19 mmol/L, bicarbonate 4.3 mmol/L), acute renal failure (creatinine 440 µmol/L, urea 31.8 mmol/L) and hepatic cytolysis (ASAT 553 UI/L, ALAT 289 UI/L). The Simplified Acute Physiology Score II (SAPS II) was 46 at admission to the ICU. A vascular filling was initiated with up to three liters of isotonic crystalloid and empiric antibiotic therapy was started with cefotaxime, daptomycin and a dose of amikacin, a usual antibiotic regimen in France for a patient with undocumented sepsis from a chronic infected wound.

**Fig. 1:**
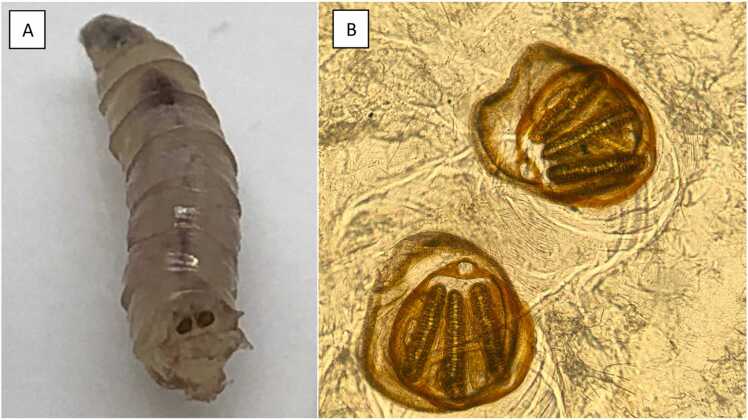
Larva identification: larva extracted from the wound (A) and respiratory stigmata from *Calliphora vomitaria* observed after Arabic gum and chloral treatment under magnification X 100 (B).

The scalp wound was carefully cleaned and the maggots in the auditory canals were evacuated under otoscopic control by an otorhinolaryngology specialist. They were sent to the parasitology laboratory (Fig. 1**A**). Based on the morphology of the respiratory stigmata, the larvae were identified as *Calliphora vomitaria* (Fig. 1**B**)*.*

**Fig. 2:**
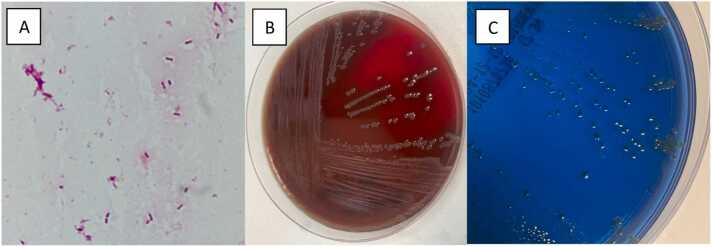
Gram staining of the aerobic blood culture showing the Gram-negative bacilli of *Ignatzschineria larvae* (panel A) and aspects of small translucent colonies after 24 h incubation of COS agar medium (panel B) and Drigalski agar medium (panel C).

Blood culture (BD BACTEC Plus Aerobic/F Culture Vials and BD BACTEC Lytic/10 Anaerobic/F Culture Vials, Becton-Dickinson, Rungis, France) collected from a peripheral site before any antibiotic perfusion grew after 15 h of incubation. Gram staining showed Gram-negative bacilli on the aerobic (Fig. 2**A**) and anaerobic bottles and Gram-positive cocci on the anaerobic bottle. GeneXpert MRSA/SA BC test (Cepheid, USA) performed on the anaerobic vial quickly identified Gram-positive cocci as methicillin sensitive *Staphylococcus aureus*. After 24 h of incubation, subculture of the aerobic vial on Columbia agar supplemented with 5% sheep blood (COS, bioMerieux, Marcy l′Etoile, France) and on Drigalski agar (bioMerieux, Marcy l′Etoile, France) under aerobic conditions grew small translucent colonies (Figs. 2**B and** 2**C**). Subculture of the anaerobic vial on Columbia agar supplemented with 5% sheep blood (COS, bioMérieux, Marcy l′Etoile, France) under anaerobic conditions, grew only white colonies typical of *S. aureus*.

*Ignatzschineria larvae* on the aerobic vial, and *Staphylococcus aureus* on the anaerobic vial were identified through Matrix Assisted Laser Desorption Ionization - Time Of Flight mass spectrometry (MALDI-TOF Biotyper Sirius; Bruker Daltonics, Bremen, Germany) with an identification score of 2.39 and 2.10 respectively. Antimicrobial susceptibility testing was performed by the disc diffusion method (I2A, Montpellier, France) and E-tests strips (bioMerieux/Thermofisher) on *Mueller-Hinton* medium. The *Ignatzschineria larvae* strain was susceptible to penicillins, carbapenems and cephalosporins and did not produce any detectable beta-lactamase. It was also susceptible to aminoglycosides and cotrimoxazole but resistant to all tested quinolones and tetracycline (Table 1).

**Table 1:** Antimicrobial susceptibility testing of the *Ignatzschineria larvae* isolate.

Antibiotic family	Antibiotic	MIC (mg/L)
Beta-lactam	Amoxicillin	0.094
	Amoxicillin/Clavulanate	0.094
	Piperacillin	0.016
	Piperacillin/Tazobactam	0.016
	Cefotaxime	0.047
	Meropenem	0.064
Aminoglycoside	Gentamicin	0.75
	Tobramycin	1
	Amikacin	4
Fluoroquinolone	Levofloxacin	3
	Ciprofloxacin	1.5
Tetracycline	Tetracycline	24
	Minocycline	2
	Tigecycline	0.19
Other	Cotrimoxazole	0.047
	Fosfomycin	128

MIC: Minimal Inhibitor Concentration

Whole genome sequencing was performed using Illumina technology. Bacterial strain DNA was extracted using the EZ1 DNA tissue kit (QIAgen, Hilden, Germany). DNA libraries were prepared with the Illumina NextEra kit (Illumina, San Diego, CA, United States) and were sequenced on an Illumina MiniSeq platform with a target sequencing depth above 50X. Species identification and detection of cross-contaminations were verified using the software MetaPhlAn v4.0.2. De novo genome assemblies were conducted using SPAdes v3.11.1 and the assembly quality was checked using QUAST v5.0.2. Genome annotation was carried out using the software Prokka v1.14.0. Resistance genes were identified using the Diamond v0.9.22 software, and ResFinder4 database. Whole genome sequencing of the strain confirmed its identification: *Ignatzschineria larvae*. The quinolone resistance determining regions (QRDR) of the DNA gyrase genes gyrA and gyrB, and of the DNA topoisomerase IV genes parC and parE, were studied. Mutations in gyrA: S83L and in parC: E84l were identified [1], explaining fluoroquinolone resistance on phenotypic antibiotic susceptibility testing. Likewise, the presence of tet (B) determinant explained the phenotypic resistance described for tetracycline and minocycline [2].

Antibiotic therapy was then changed to trimethoprim-sulfamethoxazole (oral) and oxacillin (intravenous) for a total of 7 and 14 days respectively. The clinical evolution was rapidly favorable, with normalization of all parameters within 48 h. A clinical and paraclinical examination (transthoracic echocardiography, chest-abdomen-pelvis Computed Tomography scan, and cerebral Magnetic Resonance Imaging) revealed no lesions secondary to bacteremia. Control blood cultures taken the day after the start of antibiotic therapy came back negative. The patient did not relapse on discontinuation of antibiotic therapy.

## Discussion

*Ignatzschineria spp.* is a Gram-negative, obligate aerobic, non-fermenting, non-spore-forming rod. It is mostly recovered as a commensal bacterium from the digestive tract of flies’ maggots of the *Sarcophagidae* family. These flies are ubiquitous and are frequently responsible for larval infestations in animals, notably ruminants and cattle, but more rarely in humans.

Identification of *Ignatzschineria* bacteria has been challenging for the past 20 years: the genus originally named *Schineria* was first identified in 2001 [3]*,* then renamed in 2007: *Ignatzschineria* [4]*.* Three species, namely *I. larvae*, *I. ureiclastica* and *I. indica*, have been isolated only in 2011, so that all *Ignatzschineria larvae* or *Schineria larvae* infections prior to 2011 may be due to either of the three species [5]. A fourth one, *I. cameli*, was discovered in 2018 in a dromedary wound, and has never been reported in human infection so far [6].

*I. indica* is the subspecies most often implicated in human infections, especially in the United States but infections with *I. larvae* and *I. ureiclastica* have also been described, notably in Europe. MALDI-TOF identification was unreliable until recently, with the first MALDI-TOF identification of *I. larvae* reported in 2023 [7], [8], with some difficulties in differentiating *I. indica, I. ureiclastica* and *I. larvae* [9], [10]. Thus, 16S rRNA sequencing is often used, which is still sometimes not precise enough to distinguish *I. larvae* from *I. ureiclastica* [10], [11]. Whole Genome Sequencing (WGS) was then sometimes used to explore further [12], [13]. DiFranza et al. even proposed that *I. larvae* and *I. ureiclastica* may represent a unique species due to 258-SNP pairwise distance through WGS.

The first *Ignatzschineria spp.* bacteremia were described in 2007 [14], [15]. Since then, other infections have been described, but they remain rare. Due to identification challenges, only confirmed cases of bacteremia caused by *I. larvae* have been reported to date [7], [8], [12], [16], [17], [18], [19], [20], with our case being the 9th (confirmed by WGS). Data is scarce regarding antibiotic-susceptibility, implicated maggots and risk factors. The main characteristics of these cases are listed in Table 2.

**Table 2:** Summary of Case Reports of *I. larvae* human infections.

**Case**	**Grasland et al.**	**Berthod et al.**	**Nadrah et al.**	**DiFranza et al.**	**Johnson et al.**	**Demurtas et al.**	**Gigante et al.**	**Gamboa et al.**	**Badoual et al.**
**Year**	2020	2020	2021	2021	2023	2023	2025	2026	2026
**Country**	France	Switzerland	Slovenia	USA	USA	Switzerland	Italy	USA	France
**Sex, Age (Yr)**	Male, 50	Male, 82	Male, 18	Male, 58	Male, 5 A	Male, 83	Male, 81	Male, 61	Male, 55
**Co-morbidities**	Alcohol abuse	Type 2 diabetes	0	Alcohol & drug abuse	Alcoholic cirrhosis	Arteriopathy	Obesity, COBP	Alcoholic cirrhosis	0
**Context**	Homeless	Poor social conditions	Migrant, homeless	Poor social conditions	Poor social conditions	Poor social conditions	Poor social conditions	Poor social conditions	Poor social conditions
**Identification**	MALDI: fail 16S: *I. larvae*	MALDI: fail 16S: *I. larvae*	MALDI: fail 16S: *I. larvae*	MALDI: fail 16S: *I. larvae* / *I. ureiclastica* NGS: *I. larvae*	MALDI: *I. larvae*	MALDI: *I. larvae*	MALDI: fail 16S: *I. larvae*	NR	MALDI: *I. larvae* NGS: *I. larvae*
**WGS**	No	No	No	Yes	No	No	No	No	Yes
**Phylogenic tree**	No	No	Yes (16S)	Yes (NGS)	No	No	No	No	Yes (NGS)
**Antibiogram type**	Solid	NR	E-tests	Liquid	NR	NR	Liquid	NR	E-tests
**Reported antibiotic sensitivity**	BL, FQ, CMX, Aminoside	NR	TZP, CPX, Imipenem, Ceftazidime	LVX	BL, AMK, FQ, CMX, Meropenem, Tobramycin	Ertapenem, LVX, CMX,	BL, AMK, Tigecyclin, Colistine	NR	BL, CMX, AMK, Tigecycline
**Reported antibiotic resistance**	Fosfomycin	NR	0	Tetracycline	0	0	0	NR	LVX, CPX, Tetracycline, Minocycline, Fosfomycin
**Maggots**	**Yes**	**Yes**	**Yes**	**Yes**	**Yes**	**Yes**	**Yes**	**Yes**	**Yes**
**Fly identification**	NR	NR	*Lucilia*	NR	NR	NR	NR	NR	*Calliphora vomitoria*
**Outcome**	**Alive**	**Alive**	**Alive**	**Alive**	**Alive**	**Alive**	**Alive**	**Alive**	**Alive**
**Portal of entry**	Chronic lower limb wounds	Chronic lower limb wound	Chronic lower limb wounds	Chronic lower limb ulcer	Chronic lower limb ulcer	Chronic lower limb ulcer	Chronic lower limb ulcer	Lower limb and urethral wounds	Chronic scalp wound
**Osteitis**	Yes	Yes	No	No	No	No	No	No	No
**Co-infection**	No	*P.mirabilis* + *P.stuartii*	No	No	No	No	No	*S. aureus* + *C. glutamicum*	*MSSA*
**Sepsis**	Yes	No	Yes	Yes	No	No	No	Yes	Yes
**ICU**	No	No	No	No	No	No	No	Yes	Yes
**Fluid bolus**	No	No	Yes	Yes	No	No	No	NR	Yes
**Surgery**	Amputation	Debridement	Debridement	No	No	Debridement	No		No
**Antibiotics**	Ceftriaxone + gentamycin → amoxicillin	Amoxicillin + Clavulanic Acid → TZP → CMX	Cloxacillin + CPX → Tigecycline + VAN → Imipenem + VAN	TZP + VAN + MZT → LVX	Linezolid, cefepime, metronidazole → linezolid + CFX	Ertapenem → CMX	TZP	VAN + Cefepime →VAN + CTX	Cefotaxime + Daptomycin + AMK → Oxacillin + CTX
**ATB duration**	21 days	6 weeks	10 days	7 days	14 days	3 months	NR	28 days	14 days

MALDI: MALDI-TOF; NR: Not Reported; MSSA: Meticillin-Susceptible *S.aureus*; COBP: Chronic Obstructive Pulmonary Disease; ICU: Intensive Care Unit

TZP: Piperacillin-Tazobactam; MTZ: Metronidazole; CPX: Ciprofloxacin; VAN: Vancomycin; CMX: Cotrimoxazole; LVX: Levofloxacin; AMK: Amikacin; BL: Betalactamin; FQ: Fluoroquinolone;

*Ignatzschineria larvae* bacteremia are always associated with larval infestation of a wound or ulcer and unfavorable socio-economic conditions [7] and often associated with alcohol use disorders and peripheral vascular disease [7], [12], [16], [20]. Isolation and culture from wound samples is difficult because of the overlay by other larvae commensal bacteria, notably *Providencia stuartii* or *Proteus penneri* [18] and human cutaneous flora. Three cases reported osteitis, but none reported cultures from deep bone samples. Entomological diagnosis is of major interest in this type of situation as identifying the larvae can help identifying the bacteria involved in the infection*.*

Most *Ignatzschineria* strains found in the literature are sensitive to beta-lactam antibiotics, with one exception of resistance to all beta-lactams, including carbapenems in *I. larvae* or *I. ureiclastica* infection (imprecise identification) [9]. Rare beta-lactam resistances have also been described for *I. indica* [9]. No clearly identified *I. larvae* have shown resistance to beta-lactams. No antibiotic-susceptibility has been proved to distinguish the different strains: they are classically susceptible to quinolones, cyclins and cotrimoxazole. *I. larvae* and *I. ureiclastica* are constantly resistant to fosfomycin when tested, however no information regarding fosfomycin-sensitivity has been reported for *I. indica* [13]*.* Interestingly, the strain of *I. larvae* isolated from our patient presented an atypical antibiotic resistance profile with no susceptibility to all quinolones tested (levofloxacin and ciprofloxacin) as well as to tetracycline and minocycline, confirmed by WGS. The patient reported no history of antibiotic use in the previous three months, but resistance to fluoroquinolones in Gram negative rods is common in France due to the wide use of these antibiotics.

Even though *I. larvae* are classically highly susceptible to antibiotics, a wide range of antibiotic types and durations have been used, from imipenem to amoxicillin alone and from 7 days of treatment to 3 months pending on osteitis suspicion or not [7], [12], [16], [18]. All cases described had favorable outcomes.

This case is the second described case of *I. larvae* bacteremia in a patient admitted to an intensive care unit for sepsis. Both patients admitted to the ICU presented with concomitant *S. aureus* and *I. larvae* bacteremia [20]: patients with myiasis may develop infections involving both common skin bacteria and bacteria associated with myiasis. Consistent with the literature, our patient bacteremia was associated with a wound infected with maggots. While the number of cases of *Ignatzschineria* bacteremia remains rare, the actual number of cases is probably underestimated. Indeed, *Ignatzschineria* larvae has only recently been described, and the molecular identification techniques needed to routinely identify it have only been available for a few years. It is also the first case of fluoroquinolone-resistant *I. larvae* infection, combining resistance to tetracycline and fosfomycin. This underscores the need for a precise entomologic diagnosis and blood cultures to be taken systematically in cases of larval infestation by Sarcophagidae family potentially carriers of this type of commensal bacteria, and for *Ignatzschineria larvae* to be considered in the choice of probabilistic antibiotic therapy.

## Consent

The authors certify that they have obtained the patient’s informed consent to publish this report.

## Funding

This study did not receive any specific funding from commercial or non-commercial sources. The authors covered all costs associated with this research.

## CRediT authorship contribution statement

**Angèle Roudeau:** Writing – original draft, Investigation, Formal analysis, Data curation, Conceptualization. **Paul Badoual:** Writing – original draft, Investigation, Formal analysis, Data curation, Conceptualization. **Julien Le Marec:** Writing – review & editing, Writing – original draft, Supervision, Methodology. **Djamal Haouchine:** Writing – review & editing, Writing – original draft, Supervision, Resources. **Nicolas Argy:** Writing – review & editing, Writing – original draft, Supervision, Resources. **Émilie Rondinaud:** Writing – review & editing, Writing – original draft, Supervision, Methodology.

## Declaration of Competing Interest

The authors declare no competing interests.

## References

[1] Hooper D.C. (1999). Mechanisms of fluoroquinolone resistance. Drug Resist Updat.

[2] Grossman T.H. (2016). Tetracycline Antibiotics and Resistance. Cold Spring Harb Perspect Med.

[3] Tóth E., Kovács G., Schumann P., Kovács A.L., Steiner U., Halbritter A. (2001). Schineria larvae gen. nov., sp. nov., isolated from the 1st and 2nd larval stages of Wohlfahrtia magnifica (Diptera: Sarcophagidae). Int J Syst Evol Microbiol.

[4] Tóth E.M., Borsodi A.K., Euzéby J.P., Tindall B.J., Márialigeti K. (2007). Proposal to replace the illegitimate genus name Schineria Toth et al. 2001 with the genus name Ignatzschineria gen. nov. and to replace the illegitimate combination Schineria larvae Toth et al. 2001 with Ignatzschineria larvae comb. nov. Int J Syst Evol Microbiol.

[5] Gupta A.K., Dharne M.S., Rangrez A.Y., Verma P., Ghate H.V., Rohde M. (2011). Ignatzschineria indica sp. nov. and Ignatzschineria ureiclastica sp. nov., isolated from adult flesh flies (Diptera: Sarcophagidae). Int J Syst Evol Microbiol.

[6] Tsang C.C., Tang J.Y.M., Fong J.Y.H., Kinne J., Lee H.H., Joseph M. (2018). Ignatzschineria cameli sp. nov., isolated from necrotic foot tissue of dromedaries (Camelus dromedarius) and associated maggots (Wohlfahrtia species) in Dubai. Int J Syst Evol Microbiol.

[7] Demurtas S., Pareti E., Madanchi M. (2023). Ignatzschineria larvae Bacteremia in a Patient With Chronic Leg Ulcer: A Case Report and Review of the Literature. Cureus.

[8] Johnson M.L., Kennedy B., Santos P.A. (2023). A Confirmed Case of Ignatzschineria larvae Bacteremia From a Myiatic Wound Infection in Kentucky. AIM Clin Cases.

[9] Maniam K., Argentine S. (2022). A case of sepsis due to a rare carbapenem-resistant Ignatzschineria species. IDCases.

[10] Belote A., Hawkinson D., Shoemaker D.M. (2024). Myiasis as a Vector for Bacteremia: A Unique Case of Helcococcus kunzii and Ignatzschineria ureiclastica/larvae Polymicrobial Bacteremia from Myiasis. Vector Borne Zoonotic Dis.

[11] Reed K., Reynolds S.B., Smith C. (2021). The First Case of Ignatzschineria ureiclastica/larvae in the United States Presenting as a Myiatic Wound Infection. Cureus.

[12] DiFranza L.T., Annavajhala M.K., Uhlemann A.C., Green D.A. (2021). The Brief Case: A Maggot Mystery-Ignatzschineria larvae Sepsis Secondary to an Infested Wound. J Clin Microbiol.

[13] Le Brun C., Gombert M., Robert S., Mercier E., Lanotte P. (2015). Association of Necrotizing Wounds Colonized by Maggots with Ignatzschineria-Associated Septicemia. Emerg Infect Dis.

[14] Maurin M., Delbano J.N., Mackaya L., Colomb H., Guier C., Mandjee A. (2007). Human infection with Schineria iarvae. Emerg Infect Dis.

[15] Roudiere L., Jean-Pierre H., Comte C., Zorgniotti I., Marchandin H., Jumas-Bilak E. (2007). Isolation of Schineria sp. from a man. Emerg Infect Dis.

[16] Grasland O., Donnio P.Y., Jego P., Tattevin P., Alix L. (2020). [Ignatzschineria larvae bacteremia and osteitis on a chronic wound infested by maggots]. Med Mal Infect.

[17] Berthod D., Duss F.R., Palazzuolo M., Eyer M., Onya O., Aellen S. (2020). [Myases from here and elsewhere: pseudo-furonculosis and Ignatzschineria larvae bacteremia]. Rev Med Suisse.

[18] Nadrah K., Biškup U.G., Špik V.C., Premru M.M., Šoba B. (2021). Ignatzschineria larvae Bacteremia Following Lucilia sp. Myiasis in an Irregular Migrant: A Case Report. Korean J Parasitol.

[19] Gigante P., Arcari G., Ossola D., Pennella B., Guasti L., Novazzi F. (2025). Maggot-associated Ignatzschineria larvae bacteremia: a case report. ASM Case Rep.

[20] Gamboa S., Parsons C., Shenvi C. (2026). Maggot infestation leading to Ignatzschineria larvae bacteremia and bladder outlet obstruction. Am J Emerg Med.

